# 

**Published:** 1853-04

**Authors:** 


					﻿THE
WESTERN JOURNAL
or
MEDICINE AND SURGERY.
EDITORS.
LUNSFORD P. YANDELL, M. D.
PROFESSOR OF PHYSIOLOGY AND PATHOLOGICAL ANATOMY
IN THE UNIVERSITY OF LOUISVILLE.
AND
THEODORE S. BELL, M.D.
Receipts of Medical Journal for April, 1853.
Dr. T. B. Gibson, Peters Creek, Ky., -	-	-	-	$5 CO
Dr. J. Clayton, Christiansburg, Ky., -	-	-	20 00
Dr. B. Craven, Fairmont, Mo.,....................................5	00
Dr. J. Hale, Fordsville, Ky., ...................................5	00
Dr. Eve, Augusta, Geo.,..........................................5	00
Dr. J. W. Boyle, Ricreall, Oregon, .	-	-	-	5 00
Dr. Gaines & Henry, Hopkinsville, Ky.,	-	5 00
Dr. R. R. Fitzhue, New Hope, Miss., .	*	5 00
Dr. J. A. Walton, Clarksville, Aik., -	.	.	10 00
Dr. J. is. Callings, Jamestown, Ind., -	-	-	.	5 00
Dr. J. Cook, Newport, Ind.,.....................................10	00
Dr. Burdett, Lancaster, Ky ,	-	-	.	-	.	.	5 00
Dr. Geo. W. Forman, High Gioveffty., *	-	-	.	5 00
Dr. Hunt, Louisville, Ky.,......................................10	00
Dr. R. W. Huey, Raleigh, Miss,, -	-	.	-	15 00
The Westers Journal or Medicine and Surgery is published monthly by
the undersigned, at the corner of Main and Fifth streets, Louisville, at $5 per
annum, payable in advance. Each number contains 96 pages, making two vol*
urnes in the year of more than 550 pages each.
Contributions to its pages by the Physicians of the Valley of the Mississippi
addressed to the Editors, are respectfully solicited.
Letters on business, io be addressed,postage paid, to the publishers.
‘ PRENTICE At HENDERSON
				

